# Research on Finger Vein Image Segmentation and Blood Sampling Point Location in Automatic Blood Collection

**DOI:** 10.3390/s21010132

**Published:** 2020-12-28

**Authors:** Xi Li, Zhangyong Li, Dewei Yang, Lisha Zhong, Lian Huang, Jinzhao Lin

**Affiliations:** 1 School of Communication and Information Engineering, Chongqing University of Posts and Telecommunications, Chongqing 400065, China; lixixyz@126.com (X.L.); dewei@163.com (D.Y.); lisa@126.com (L.Z.); hlcqsxxy@163.com (L.H.); 2 Foundation Department, Chongqing Medical and Pharmaceutical College, Chongqing 401331, China; 3 School of Bioinformatics, Chongqing University of Posts and Telecommunications, Chongqing 400065, China; lizy@cqupt.edu.cn

**Keywords:** finger vein, Gabor, Gaussian mixture model, image segmentation

## Abstract

In the fingertip blood automatic sampling process, when the blood sampling point in the fingertip venous area, it will greatly increase the amount of bleeding without being squeezed. In order to accurately locate the blood sampling point in the venous area, we propose a new finger vein image segmentation approach basing on Gabor transform and Gaussian mixed model (GMM). Firstly, Gabor filter parameter can be set adaptively according to the differential excitation of image and we use the local binary pattern (LBP) to fuse the same-scale and multi-orientation Gabor features of the image. Then, finger vein image segmentation is achieved by Gabor-GMM system and optimized by the max flow min cut method which is based on the relative entropy of the foreground and the background. Finally, the blood sampling point can be localized with corner detection. The experimental results show that the proposed approach has significant performance in segmenting finger vein images which the average accuracy of segmentation images reach 91.6%.

## 1. Introduction

Due to COVID-19, the technology of fingertip blood automatic sampling has attracted extensive attention. Compared with traditional manual blood collection, the intelligent blood collection has outstanding advantages in blood collection efficiency, quality, and preventing infection of medical staff [1]. In fingertip blood sampling, because of the insufficient bleeding, the fingertip needs to be squeezed, so that the tissue fluid could penetrate into the blood mark and destroy the overall structure of white blood cells, then, the blood sample is diluted, leads to inaccurate test results [2]. Generally speaking, the accuracy of arm venous blood test is higher than that of fingertip blood [3], and if the finger venous area is selected as a blood sampling point, the amount of bleeding will be clearly increased and the accuracy of blood detection will be improved.

Image segmentation methods have made great achievements in many fields, but some gaps still exist in finger vein segmentation [4,5]. Due to the poor contrast of finger vein images, current segmentation methods cannot effectively distinguish the vein from non-venous areas [6,7]. How to locate the blood sampling point to the finger venous area is the difficulty and hotspot of current research. A large number of new methods have been proposed, which are basically divided into four categories.

Contrast-enhanced method: this method uses image enhancement algorithms to make the vein network more obvious and easier to extract. In order to enhance the contrast of finger vein images, Ezhilmaran et al. [8] improved finger vein image by interval type-2 fuzzy sets method. Amir et al. [9] adopted dual-contrast limited adaptive histogram equalization method, which is used to enhance the grayscale color intensity values. Kashif et al. [10] used a single scale retinex (SSR) filter with chromaticity preserved algorithm and Gaussian filter to enhance the low-quality finger vein images. Cao et al. [11] calculated the amplitude value of the vein image edge operator to punish the fixed neat parameter in the guided filter which makes the filter possess a better edge protection feature. These methods can well solve the problem of low contrast of finger vein images.

Energy-based method: this method uses continuous curves to represent the edges of objects. Zhang et al. [12] achieved finger vein image segmentation by minimizing the proposed region-based active contour model (ACM) which is served as an energy function, and the level set method is introduced to solve the minimization problem efficiently.

Curvature-based method: this method doesn’t require any preprocessing to detect the valley area generated by the texture pattern. Taking into account that there are always dark valleys in finger vein patterns, a curvature-based method is used to detect the center of the vein. Vasilopoulos et al. [13] utilized the enhanced maximum curvature Points (EMC) technique for finger vein pattern extraction. Wang et al. [14] used variable curvature Gabor filters to extract finger vein features that can simultaneously reflect the directional information and the curvature of the finger veins. These methods are more efficient in detecting thin veins and reserve areas.

Deep learning-base method: deep neural network framework has achieved good results in image segmentation. Reza et al. [15] used a new fully convolutional encoder-decoder model for lung segmentation and improved the state-of-the-art u-net model which introduced a pre-trained encoder, a special skip connection, and a post-processing module in the proposed architecture. It is rarely used in finger vein segmentation for the reason that there is no expert annotated dataset of the finger vein. To overcome this problem, some automatic labeling schemes have been proposed. Hou et al. [16] integrated a convolutional auto-encoder (CAE) with support vector machine (SVM) for finger vein verification. The CAE is used to learn the features from finger vein images, and the SVM is used to classify finger vein from these learned feature codes. Qin et al. [17] trained a convolutional neural network (CNN) model to predict the probability of each pixel of being foreground. The CNN learns what a finger vein pattern is by learning the difference between vein patterns and background ones. They still use the traditional modeling method to segment finger vein as training dataset, so the accuracy of segmentation still depends on the traditional modeling method.

The latest progress of research about the deep attention-based spatially recursive networks [18] may achieve good results in finger vein segmentation. It can finely recognize the visual objects with subtle appearance differences by operating two CNN streams to automatically learn to attend critical object parts, extract relevant features, and encode them into spatially expressive representations. The extraction method based on deep learning needs a large number of manual annotation samples to optimize the learning process. As far as we know, there is no annotated dataset of finger vein by experts. Therefore, we choose the traditional modeling method to explore a mathematical method suiting for finger vein image segmentation.

Gabor feature extraction which has excellent features of time-domain localization, scale change, and orientation, can simulate the visual recognition mechanism of the human eye [19] approximately, achieve the capability of multi-scale and multi-orientation description has excellent features of time-domain localization, scale change, and orientation. The cluster analysis method [20] of Gaussian mixture model can solve the problem of uncertain classification and image segmentation with complex content [21]. Therefore, the Gabor feature extraction and GMM have attracted extensive attention in the field of finger vein image segmentation [22]. But Gabor filter parameters selection needs researchers’ experience and the large amount of Gabor feature data of the image leads to long processing time and poor real-time performance.

In view of these difficulties, we a present finger vein segmentation method based on the joint decision of adaptive Gabor feature extraction and GMM. The primary contributions of our study are summarized as follows:

- To take full advantage of the differential excitation of the image, we propose an adaptive parameter setting method for Gabor filter banks.
- Dimensionality reduction on high dimensional features is researched, we adopt fusion algorithm [23,24] based on LBP to fuse the same-scale and multi-orientation Gabor features of the image.
- GMM is used to classify the features of multi-scale Gabor images after fusion and optimized with the method of max flow min cut [25,26] based on the relative entropy of the foreground and the background.

The experiments exhibit that our proposed method is effective for improving the segmentation accuracy of finger vein images and increasing the amount of bleeding without being squeezed.

## 2. Image Feature Extraction

### 2.1. Fingertip Blood Automatic Sampling Device

**Figure 1:**
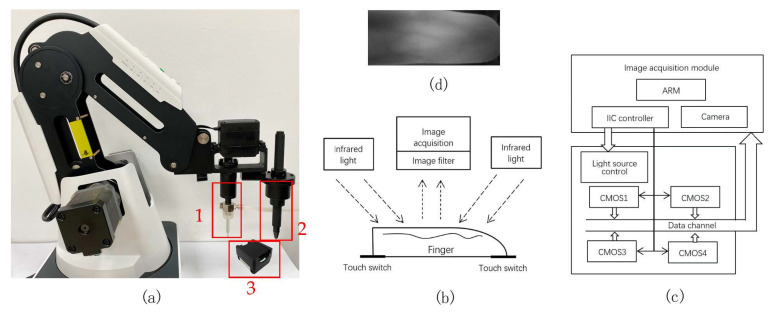
Finger vein imaging and blood sampling device. (**a**) Mechanical arm, (**b**) Reflection infrared imaging of finger-vein, (**c**) Circuit structure of imaging module, (**d**) Infrared image of finger-vein.

The fingertip blood automatic sampling device is shown in Figure 1a. It consists of a 5-DOF robotic arm, blood collection tube (red box 1), lancet (red box 2) and infrared imaging module of finger vein (red box 3) constituted. The infrared imaging module as shown in Figure 1 uses a kind of reflection imaging method. The reflective imaging facilitates the light source and sensor to be packaged together, and the equipment becomes more compact. The circuit structure of the imaging module is shown in Figure 1c. It contains 4 CMOS image sensors. When vein acquisition is performed, the IIC controller of the main control chip adjusts the intensity of the near-infrared light source in real-time according to the environment and realizes time-sharing multiplexing of data lines. The acquired finger vein image is shown in Figure 1d.

The original image of finger vein is not preprocessed, and the irrelevant image information not only increases the calculation time but also interferes with the segmentation result of the finger vein image. The blood sampling point is at the end of the finger, so we only need to segment the fingertip vein, which can improve the accuracy and speed of finger vein image segmentation.

If we don’t preprocess the original image of the finger vein, the irrelevant image information will not only increase the calculation time, but it will also interfere with the segmentation result of the finger vein image. The blood sampling point is at the end of the finger, and we only need to segment the fingertip vein, which can improve the accuracy and speed of finger vein image segmentation.

### 2.2. Adaptive Gabor Filter Parameter Setting

Gabor filter parameters include polar center frequency, orientation and window width, etc., and different filters can be obtained by changing these parameters. In addition, the information obtained in each orientation and scale can fully reflect the characteristics of the image in the frequency domain by using a set of Gabor filters in different orientations and scales, so as to filter the finger vein image. The basic Gabor function g(x,y) is expressed as follows:

(1) $$g\left(x,y\right)=\frac{l}{2\pi \sigma^{2}}e^{\frac{-\left(u^{2}+v^{2}\right)}{2\sigma^{2}}}\left(e^{j\omega u}-e^{-\omega^{2}\sigma^{2}/2}\right)$$

(2)  u=xcosθ+ysinθ,  v=−xsinθ+ycosθ

In which *x* and *y* are the pixel positions in the spatial domain, *ω* is the modulation frequency of the filter, *σ* is the standard deviation of the gaussian function on the x-axis and y-axis; *θ* is the filter direction. The relationship between ω and σ is expressed as $\sqrt{2}\;\sigma \approx \sqrt{2}\pi /\omega =W$, *W* is the time domain window width. The selection of *W* is highly important as it determines what information is going to be extracted. The filtered image I_0_ can be obtained by convoluting the original image with Gabor function [27,28], $I_{0}$ can be expressed: $I_{0}=g\left(x,y\right)*I\left(x,y\right)$.

The time-frequency window of the traditional Gabor transform is fixed. In the feature extraction of finger vein images, different regions of the image need different time-frequency windows to extract corresponding feature information, and the fixed window has a poor effect on the feature extraction of finger vein images. Finger vein images have more veins in some areas and complex frequency composition, which requires better frequency resolution [29,30], and slow changes in some areas require better spatial resolution.

**Figure 2:**
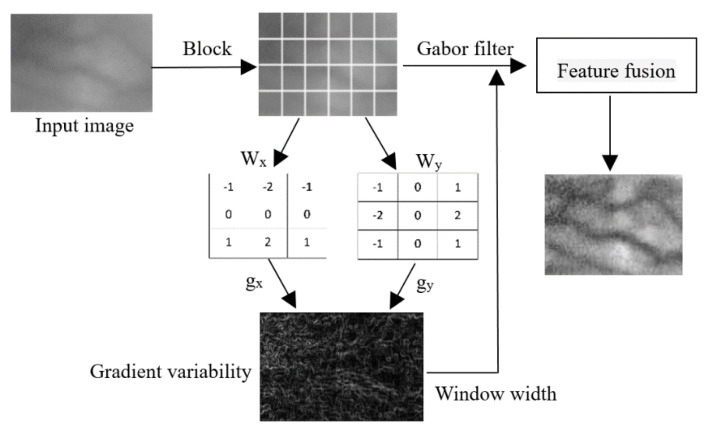
Adaptive feature extraction process.

Differential excitation [31] has the characteristics of reflecting the local changes of image. In this paper, the differential excitation is used to measure the intensity of the local image change. The adaptive parameter setting process of filter is shown in Figure 2. After dividing the finger vein image into K × L blocks, the gradient amplitude and differential excitation can be obtained by convoluting the sub image blocks with gradient operators in x and y directions, gradient operators are shown in $W_{x}$ and $W_{y}$. Then we can calculate the window width of Gabor filter bank according to the image differential excitation.

We select a vein sub image block P(u,v), and then the gradient template is convoluted with the image to obtain the gradient components $g_{x}$ and $g_{y}$ in the horizontal and vertical directions of each pixel, we get

(3) $$\{\begin{array}{c} g_{x}=P\left(u,v\right)*W_{x}\\ g_{y}=P\left(u,v\right)*W_{y} \end{array}$$

Gradient amplitude is used to describe the relative change of gray level, and the central window width *W* is calculated as

(4) $$W=\frac{\sum_{x=0}^{M}\sum_{y=0}^{N}\sqrt{g_{x}^{2}+g_{y}^{2}}/P\left(u,v\right)}{MN}$$

**Figure 3:**
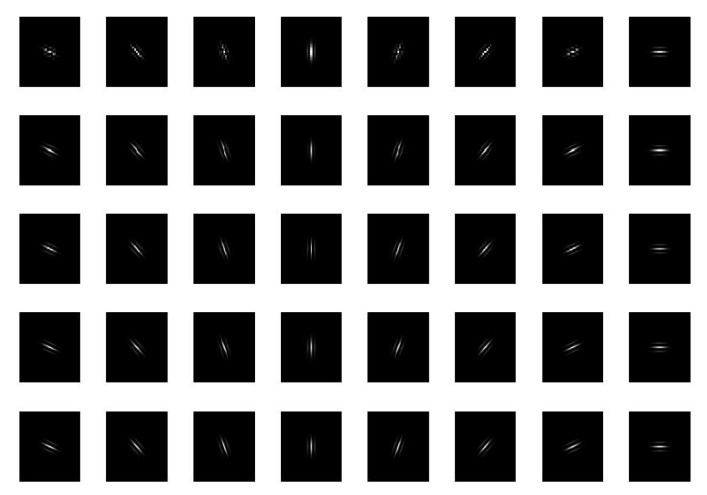
Gabor filter banks with different orientations and window widths.

According to the strong setting of the local area change, the width of the Gabor window function is set, and the sum of all the differential excitation values in the corresponding image block is divided by the number of pixels in the image block as the window width of the central window in Gabor transform. Five Gabor windows with different scales are used, in which the adaptive window width is taken as the center window, and the other four Gabor windows are selected in steps of 2 in the positive and negative directions, and the Gabor filter bank is formed in 8 orientations. As shown in Figure 3, 8 different orientations are used to extract the finger vein feature by 5 Gabor filter groups with different window widths.

### 2.3. Image Feature Fusion

We have obtained Gabor feature images with 5 scales and 8 directions, so each pixel of the image can get 40 features after Gabor transform, considering the processing time, the dimension of the data needs to be reduced, so we propose a multi-orientation fusion method [32,33] of local binary mode. The original Gabor feature of the image is expressed as *P(s,t)*, *s* ∈ (1,…,8), *t* ∈ (1,…,4). The average value of the characteristic amplitudes of the 8 orientations on each scale of the image pixel is used as the threshold to binarize the characteristic amplitudes of each orientation.

(5) $$avg=(P_{1,t}+P_{2,t}{}_{.}\ldots +P_{8,t})/8$$

The 8 orientations amplitude feature fusion is expressed as:

(6) $$T\left(s\right)=\left(P_{s,t}-\mathrm{avg}\right)=\{\;\begin{array}{c} 1,\;\;P_{s,t}-\mathrm{avg}>0\\ 0,\;P_{s,t}-\mathrm{avg}\leq 0 \end{array}\;s\in \left(1,2,\ldots ,8\right)$$

*T(s)* = 1, 2, …, 8 is sorted to get an 8-bit binary number *p*, and each 8-bit binary number is assigned a weight of $2^{p}$. Therefore, the decimal form of the fusion code can be expressed as:

(7) $$F_{u}\left(z\right)=\sum_{s=1}^{8}\left(P_{s,t}-avg\right)2^{p}$$

Algorithm 1 summarizes finger vein image adaptive Gabor filter and image feature fusion algorithm.

**Algorithm 1** Adaptive Gabor filter and image feature fusion.**Input**: Original image of finger vein: *I***Step 1**: Extract the region of interest of the image *I*, add 0 to the edge of the image to transform its size to 256 × 256.**Step 2**: Divide the image into 16 × 16 sub-blocks and then obtain the gabor function window width of each sub-block.  **for** each row *u*∈1, 2, …, 16 **do**   **for** each column *v*∈1, 2, …, 16 **do**    Compute the gradient components in X and Y directions per Equation (3).    Obtain the window width of Gabor filter per Equation 4.   **end**  **end****Step 3**: Gabor transform for each sub-block image:$\;I_{n}^{'}=g_{n}\left(x,y\right)*I_{n}\left(x,y\right),$ and obtain Gabor transform of the whole image.**Step 4**: Fuse the Gabor image features in 8 orientations with the same gabor transform size per Equation (3).**Output**: Finger vein feature fusion image: $F_{u}\left(z\right).$

**Figure 4:**
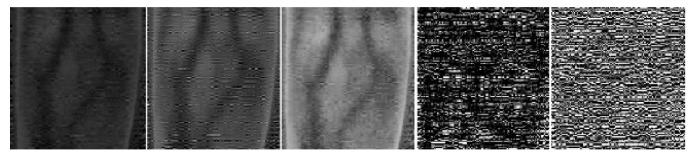
Image feature fusion.

After image fusion, feature fusion images on five scales can be obtained, as shown in Figure 4, and each image contains all Gabor orientation features.

## 3. Finger Vein Image Segmentation

After fusion of finger vein image features, we obtain the pixel point multi-scale transformation feature. We use $Fu,v=\left\{f_{1},f_{2}\ldots f_{L}\right\}$ to represent the feature of the finger vein image at point (*u*,v), where L=M×K, and the pixel point is clustered and segmented by the feature information. According to the Markov random field energy functional [34,35] definition, the energy function in the finger vein image is defined as:

(8) $$E\left(\gamma \right)=\sum_{p\in F}R_{p}\left(\gamma \right)+\beta \sum_{a,b\in N}S_{a,b}\left(\gamma \right)$$

where *p* is the vein feature corresponding to the pixel in the image after multi-scale transformation, *a* and *b* are the neighboring regions; *γ* is the segmentation label of the foreground and background, *γ* = 1 indicates the foreground, *γ* = 0 indicates the background; *N* is a collection of all adjacent pixels in the image. The first term of formula 8 describes the regional information of the image, which indicates the similarity of pixels belonging to the foreground or background; the second term is used to evaluate the penalty value when adjacent pixels *a* and *b* belong to different label sets; *β* is the weight factor, the target with a single shape and a concentrated area has a larger value, and A smaller weight factor is more suitable for targets with complex and relatively discrete local details.

The GMM probability distribution model has robustness and accuracy for image description. GMM modeling is used to describe the distribution of multi-scale vein features. In this case, we have:

(9) Rp(γ)=  ∑k=1K ρk(∏l=0N−1δτ )1N

(10) $$\delta ={\left(2\pi \sigma_{k,l\left(\gamma \right)}^{2}\right)}^{-\frac{1}{2}}$$

(11) $$\tau =\exp\left(-2\frac{{\left(p-u_{k,l\left(\gamma \right)}\right)}^{2}}{\sigma_{k,l\left(\gamma \right)}^{2}}\right)$$

In the above Equations (9)–(11), $\sigma_{k,l\left(\gamma \right)}^{2}$ and $u_{k,l\left(\gamma \right)}$ are the mean and the variance of the corresponding Gabor feature when the product of the scale and direction of the *k*th Gaussian part is *l*, when *p* is 1, the *l*th mean and variance of the *k*th Gaussian part of the foreground is taken, otherwise, the *l*th mean and variance of the background are taken. *K* is the number of cluster centers; $\rho_{k}$ is the weight of the *k* Gaussian component part of GMM, which reflects the feature contribution of the *k* Gaussian part, and its initial value is shown in formula (12).

(12) $$J=\sum_{j=1}^{k}\sum_{x_{i}\epsilon c_{k}}\|x_{i}-c_{k}\|{}^{2}$$

(13) $$c_{k}=\frac{1}{Q_{k}}\sum_{X\epsilon Q_{k}}X$$

where $c_{k}$ is the center of the *k*th cluster, $Q_{k}$ is the number of data objects in the *k*th category, and $x_{i}$ is the *i*th data point. $x_{i}-c_{k}$ represents the Euclidean distance between the *i*th object and the *k*th cluster center.$\;S_{a,b}\left(\gamma \right)$ gaussian probability distribution can be expressed as follows:

(14) $$S_{a,b}\left(\gamma \right)=\left[\gamma_{a}\neq \gamma_{b}\right]\left(d{\left(a,b\right)}^{-1}\varphi \right)$$

(15) $$\varphi =\exp\left(-\eta f{\left(a,b\right)}^{2}\right)+\pi$$

where $\gamma_{a}$ and $\gamma_{b}$ are the label values of *a* and *b*; *d*(*a*,*b*) is the Euclidean distance of the pixel value of the two points *a* and *b*; f(a,b) is the vector distance calculated by the LPP method of the local preserving projection of the multi-scale and multi-orientation Gabor vein feature of the two points *a* and *b*. In order to improve the anti-noise characteristics of the image, we introduce the anti-noise constant *τ* and the segmentation edge length limit *η*, *η* is the normalization coefficient of the vein feature, and it can be calculated as follows:

(16) $$\eta ={\left(2\sum_{m,n\epsilon N}d{\left(m,n\right)}^{2}/k_{i}\right)}^{-1}$$

where $k_{i}$ is the number of image pixels, and d(m,n) is the LPP distance metric of two multi-scale features. The energy modeling of vein image segmentation is completed by GMM modeling of foreground and background.

The image after feature fusion can be transformed into a weighted graph [4,36] with two endpoints *G* = (*V*, *E*). Where *V* is the set of image pixels and endpoints *(s, t*); *E* is the set of edges, which includes the weighted similar edges of the pixels belonging to the foreground and background, and the penalty weights between the pixels and the neighboring edges.

**Figure 5:**
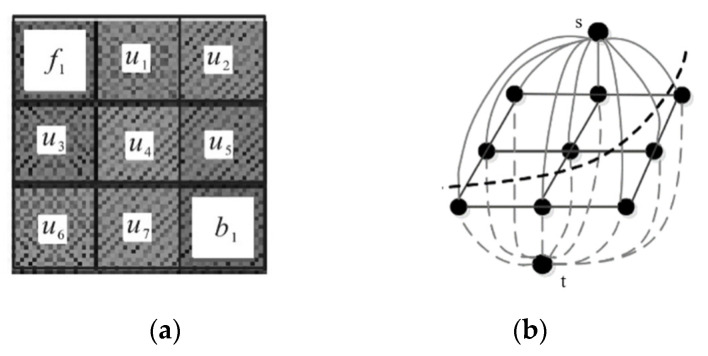
Max flow min cut. (**a**) Pixel distribution of vein image, (**b**) Dividing line.

Figure 5a is the image after feature fusion, where *f*_1_ is the foreground marker point, and *b*_1_ is the background marker point, and the weighted graph model is established through GMM. The edge between the point and the endpoint (*s*, *t*) in Figure 5b indicates the degree of similarity with the foreground or background. The upper half of the edge represents the similarity between the pixels in the vein image and the foreground [37,38], and the lower half part of the edge represents the similarity with the background. After the weighted graph is established, the global optimal graph cut is performed through the max-flow min-cut to obtain the segmentation curve shown in Figure 5b.

## 4. Location of Blood Sampling Point

After the venous area is segmented, we need to determine the coordinates of the blood sampling point. The vein junction is used as a blood sampling point, and the amount of blood we collect will be more, so we use the Harris corner detection algorithm to detect the corner points of the venous network in the target area and select the vein intersection as the blood sampling point. We first calculate the matrix *M* related to the gradient autocorrelation function in the horizontal and vertical directions of the image and its two eigenvalues. The eigenvalue of the matrix *M* is the first-order curvature of the autocorrelation function. If both curvature values exceed the threshold, the point can be regarded as a corner point. The translation amount of the image window is (*u, v*) to produce a grayscale change as E(u,v)

(17) $$E\left(u,v\right)=\sum_{x,y}w\left(x,y\right){\left[I\left(x+u,y+v\right)-I\left(x,y\right)\right]}^{2}$$

where w(x,y) window function adopts Gaussian function, I(x,y) is the gray value of the image, and the local small movement of the window can be calculated approximately as follows

(18) $$E\left(u,v\right)≅\left[u,\;v\right]M\left[\begin{array}{c} u\\ v \end{array}\right]$$

where *M* is a 2 × 2 matrix, which can be obtained from the image derivative:

(19) $$M=\sum_{x,y}w\left(x,y\right)\left[\begin{array}{cc} I_{x}^{2} & I_{x}I_{y}\\ I_{x}I_{y} & I^{2} \end{array}\right]$$

The corner point is determined by calculating the gray change of the target pixel in any direction and the corner response function of the target pixel. When the corner response function value of the target pixel is greater than a given threshold and local maximum value, we regard the pixels as corner points.

**Figure 6:**
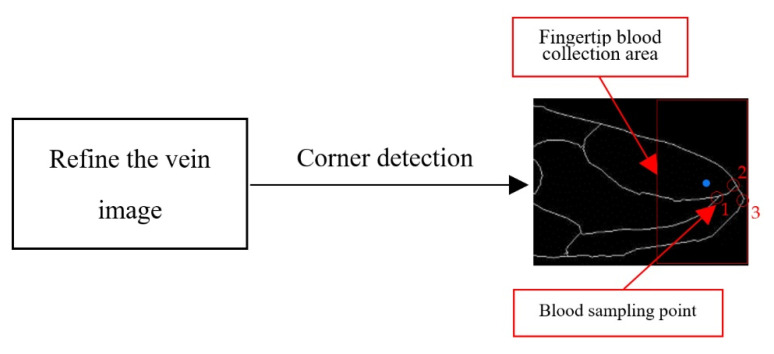
Location of blood sampling point.

There are many burrs after segmentation of the finger vein image, and the corner detection [39] is prone to errors when identifying the blood sampling point. After the vein network is refined, it can effectively reduce the burr phenomenon, and the accuracy of identifying the blood sampling point is higher. The refined vein image is shown in Figure 6.

We select a 100 × 200 rectangular box near the fingertips as the blood collection area, and calculate the center pixel coordinates of the blood collection area as p(x,y), as shown by the blue dot in Figure 6. According to the principle of corner recognition, three corner points 1, 2, 3 are identified in the blood collection area, and we select the corner point 1 which is closest to p(x,y) as the blood collection point.it is shown in the circle position in Figure 6.

## 5. Result and Discussion

The experiments were operated using matlab 2018a in a computer with i7-8700CPU and 8 GB RAM. Firstly, the region of interest (ROI) image is extracted, and then we add 0 to the edge of the finger vein image to transform the cropped image to a size of 256 × 256. Furthermore, the image is divided into 16 × 16 blocks and it adaptively sets the gabor filter bank parameters according to the differential excitation. Finally, the finger vein image is segmented by the Gabor-GMM system which is proposed in this paper.

We use self-collected finger vein infrared images and the finger vein database of the University of Technology Malaysia (FV-USM) to verify. FV-USM were collected from 123 volunteers comprising of 83 males and 40 females, who were staff and students of University Sains Malaysia. The age of the subject ranged from 20 to 52 years old. Every subject provided four fingers: left index, left middle, right index, and right middle fingers resulting in a total of 492 finger classes obtained. The captured finger images provided two important features: the geometry and the vein pattern. Each finger was captured six times in one session and each individual participated in two sessions, separated by more than two weeks’ time, and a total of 5904 (123 × 4 × 6) images were collected. The spatial and depth resolution of the captured finger images were 640 × 480 and 256 grey levels.

**Figure 7:**
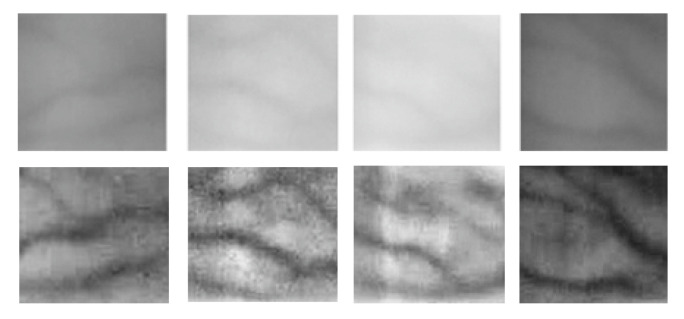
Gabor filter and feature fusion.

Before segmenting the finger vein images, we use algorithm 1 to process the original finger vein image. As can be seen from Figure 7, it is difficult to identify the venous area from the original image and the vein pattern becomes more prominent through Gabor filtering and feature fusion.

In order to estimate the performance of the proposed model on finger vein image segmentation, the proposed method is compared with other well-known methods.

**Figure 8:**
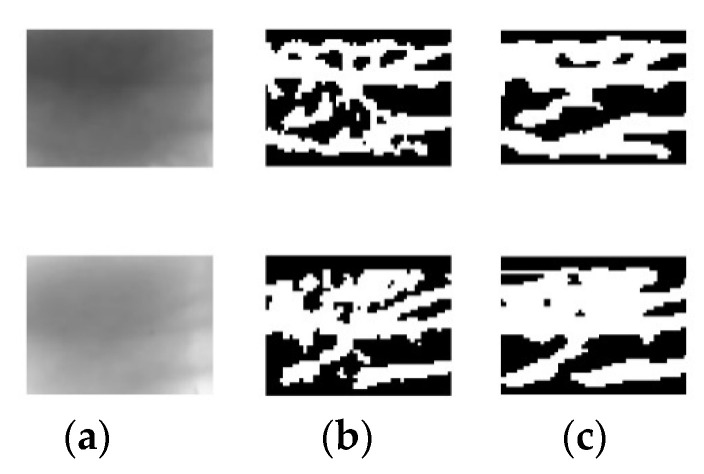
Extracted results of finger vein images; (**a**) original images; (**b**) finger vein feature extracted by combination scheme; (**c**) finger vein feature extracted by the CNN.

Qin used CNN to extract vein features [17]. MC, WLD, gabor filter, and other methods are combined to segment finger vein image, and then the segmented image is used as a training dataset. This scheme is used to automatically discard the ambiguous region and to label the pixels of the clear region as foreground or background. As can be seen from Figure 8, this method can extract smooth vein networks. However, this simple labeling method also brings serious side effects and reduces the ability of the network to extract detailed features. It reveals that the network is not adept at distinguishing the blurred area and easy to lose vein networks. The above analysis indicates that the automatic annotation method still faces challenges and greatly affects the accuracy of the network.

The challenge of deep learning methods applied in finger vein image segmentation is the lack of annotated data. [30] divided the finger vein image into a large amount of blocks with fixed size, and each block could be labeled as venous and non-venous area to conduct binary classification. This method could effectively increase the training data but will degrade the segmentation efficiency of the network.

Weber local descriptor (WLD) [14] is a simple and powerful local descriptor in which differential excitation is redefined by bringing in sobel operator, and it can increase the discrimination of edge-texture. Meanwhile, the gradient orientation is replaced by double modified finite radon transform orientation, to obtain a discriminative line feature. As such, it also effectively improves the recognition performance. Double Gabor weber local descriptor (DGWLD) [40] used gradient orientation, which is replaced by double Gabor orientation to reduce the influence of translation and rotation, and a feature cross-matching algorithm is used to give further improvement on the recognition rate. Maximum curvature (MC) [41] is to look in the transverse profiles of the image for the maximum curvatures. In the finger vein image captured by the infrared sensor, the vein is darker than the neighboring band. Hence the MC method takes advantage of the fact that the transverse profile of a finger around seems like a dent, the central position of the veins can be obtained by calculating local maximum curvatures in cross-sectional profiles, and the connection of all the points of all the profiles forms the vein line.

To further verify the effectiveness of the segmentation method proposed in this paper, we evaluate the segmentation performance of the method by 150 finger vein images which are demarcated manually as the ground truth. The Jaccard Similarity (JS) and mean bias error (MBE) are taken as the evaluation index. The JS and BME are defined as follows:

(20) $$\mathrm{JS}=\frac{R_{S}\cap T_{S}}{R_{S}\cup T_{S}}\;$$

(21) $$\mathrm{MBE}=\frac{R_{S}-T_{S}}{R_{S}}$$

where $R_{S}$ is the object region given by the ground truth, $T_{S}$ is the object region given by the segmentation methods. JS describes the segmentation accuracy and MBE represents the segmentation error rate. The average value of JS and MBE is calculated with 150 finger vein images.

**Figure 9:**
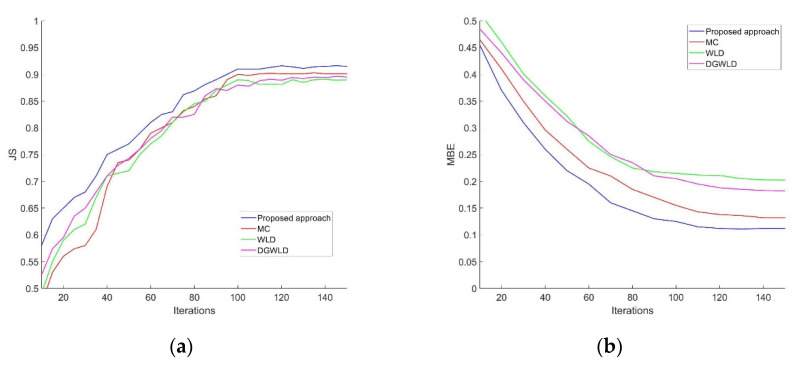
The Jaccard similarity and mean biased error. (**a**) Trend of segmentation accuracy, (**b**) Trend of segmentation error rate.

Figure 9a,b respectively show the trend of segmentation accuracy and segmentation error rate under the increasing number of iterations. As can be seen from Figure 9, the segmentation model based on our method achieves the best performance on both JS and MBE. Experiment results show that the proposed segmentation method can extract vein network from finger vein image reliably.

**Figure 10:**
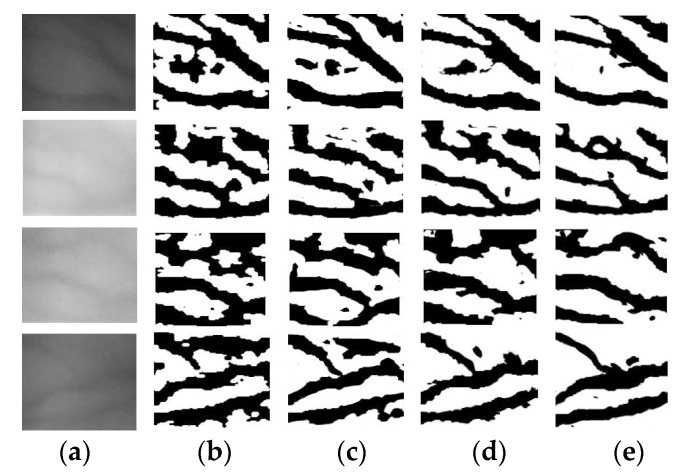
Finger vein image segmentation results by different algorithms. (**a**) Original images, (**b**–**e**) Segmentation results of WLD, DGWLD, MC, and proposed method.

Figure 10 shows the comparison results of different methods in finger vein image segmentation. Refs. [14,40] transform the gray distribution of the image to make the venous area easier to distinguish. However, low-contrast area is also easily mistaken for venous network, as shown in Figure 10b,c. Ref. [41] identifies the vein network by properties of curvature, which can solve the problem that image gray transform generates fake vein networks. Nevertheless, the method of [41] is so susceptible to noise that it could seriously degrade the final segmentation result, as shown in Figure 10d.

Through the above discussion, it is obvious that the vein image segmented by the proposed method can suppress the noise and improve the smoothness of the boundary effectively. The quantitative results are shown in Table 1. The average time-consuming of this method is slightly higher than other methods and the segmentation accuracy is significantly improved.

## 6. Conclusions

In finger vein image segmentation, image preprocessing, feature extraction and classifier design method have a great influence on the accuracy of image segmentation. Deep learning method has achieved a good result in image segmentation, and due to the lack of annotated dataset of the finger vein, the accuracy of finger vein segmentation is comparatively low. The methods of WLD and DGWLD are easy to mistake low-contrast area for the venous network; MC method is susceptible to noise and it could seriously degrade the final segmentation result.

This paper makes improvements from the perspective of vein image feature extraction, adaptively sets the parameters of the Gabor filter bank according to the local differential excitation of the image, dynamically adjusts the spatial frequency resolution to obtain more feature details of the pixels, and innovatively proposes the Gabor filter bank and GMM model joint decision finger vein feature classification algorithm that based on the relative entropy combined with the image segmentation method of max-flow min-cut to realize the global optimization of finger vein image segmentation, therefore the boundary continuity, smoothness and visual consistency of the target area of image segmentation are better, and more vein feature details can be extracted by segmentation. Compared with other methods, the accuracy of finger vein image segmentation is improved when the processing time is similar. The vein image segmented by this method can meet the requirements of finger vein blood sampling point recognition and experiments have verified that it can segment the finger vein image accurately and locate the blood sampling point in fingertip blood automatic sampling quickly. It is helpful to improve the accuracy of fingertip blood detection. As future work, we would like to design a more discriminative and computationally practicable segmentation process and we will annotate the dataset of finger veins to establish a training dataset for deep learning.

## Figures and Tables

**Table 1:** Different methods of vein segmentation accuracy and time.

Methods	Accuracy	Processing Time
WLD	0.885	1.24 s
DGWLD	0.894	1.75 s
MC	0.901	1.62 s
Method of this article	0.916	1.86 s

## Data Availability

Publicly available datasets were analyzed in this study. This data can be found here: http://drfendi.com/fv_usm_database/.
